# Histone H3K18 Lactylation Contributes to Perioperative Neurocognitive Disorder Through Immune Checkpoint Lymphocyte Activation Gene 3 Mediated Microglial Pyroptosis

**DOI:** 10.1002/cns.71058

**Published:** 2026-07-27

**Authors:** Chenglong Li, Xi Gou, Yingying Zhao, Lina Zhang, Shuai Liu, Shenhui Deng, Qi Li, Sihua Qi

**Affiliations:** ^1^ Department of Anesthesiology The Fourth Affiliated Hospital of Harbin Medical University Harbin Heilongjiang China

**Keywords:** H3K18 lactylation, Lag3, microglia, perioperative neurocognitive disorder, pyroptosis

## Abstract

**Aims:**

This study investigates whether histone H3K18 lactylation (H3K18la) contributes to perioperative neurocognitive disorders (PND) by upregulating immune checkpoint Lymphocyte activation gene 3 (Lag3) and microglial pyroptosis, thereby aggravating neuroinflammation in the hippocampus.

**Methods:**

Lactate levels and H3K18la expression were measured in mouse hippocampus after surgery. H3K18la downstream targets were identified by CUT&Tag and RNA‐seq. After Lag3 silencing in BV2 cells, downstream pathways were screened and validated by RNA‐seq and in vitro assays. Glycolysis inhibitors and Lag3 antibody were used to assess cognitive function and pyroptosis‐related protein expression in postoperative mice.

**Results:**

Surgery significantly elevated hippocampal lactate and H3K18la levels in mice. Inhibiting glycolysis led to a reduction in H3K18la and mitigated cognitive impairments in the PND model mice. CUT&Tag and RNA‐seq revealed that H3K18la transcriptionally activates Lag3 by enriching at its promoter, and Lag3 blockade improved postoperative cognition. Furthermore, Lag3 upregulation triggered microglial pyroptosis via NOD‐like receptor protein 3 (NLRP3) signaling, thereby aggravating neuroinflammation and promoting PND.

**Conclusions:**

Surgical trauma‐induced increase of H3K18la in microglia promotes PND by upregulating Lag3 and triggering NLRP3‐dependent microglial pyroptosis, highlighting the H3K18la–Lag3–NLRP3 axis as a therapeutic target for PND.

## Graphical abstract

A schematic representation illustrates how histone H3K18la promotes PND by inducing microglial pyroptosis through the immune checkpoint Lag3. Following surgical trauma, systemic inflammation damages the BBB, allowing various harmful components to enter the brain. This infiltration triggers metabolic reprogramming in microglia, resulting in lactate accumulation and an increase in H3K18la levels. Elevated H3K18la enhances the transcriptional activation of the Lag3 gene, which further promotes the assembly of inflammatory bodies by activating the NLRP3 pathway. This sequence of events ultimately leads to microglial pyroptosis and the release of proinflammatory factors, exacerbating neuroinflammation and contributing to PND.

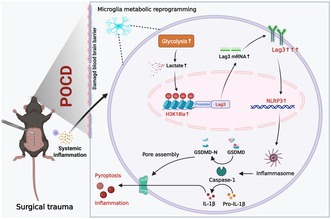

**Abbreviations**
AD: Alzheimer's disease
CNS: central nervous system
DAMPs: damage‐associated molecular patterns
H3K18la: H3K18 lactylation
IF: immunofluorescence
IFN‐γ: interferon‐γ
IL: interleukin
Lag3: lymphocyte activation gene 3
LDH: lactate dehydrogenase
LPS: lipopolysaccharide
mAb: monoclonal antibody
MAPK: mitogen‐activated protein kinase
NF‐κB: nuclear factor kappa‐B
NLRP3: NOD‐like receptor protein 3
NOR: novel object recognition
OFT: open field test
OXPHOS: oxidative phosphorylation
PCA: principal component analysis
PD: Parkinson's disease
PND: perioperative neurocognitive disorder
TLR4: Toll like receptor 4
WB: western blotting

## 1 Introduction

Perioperative neurocognitive disorder (PND) is a common and serious complication affecting the central nervous system (CNS) following surgery, particularly in elderly patients. The condition primarily manifests as memory loss, inattention, decreased learning ability, and impairment of executive function [1]. The incidence of PND ranges from 10% to 54%, significantly impacting patient prognosis. This condition leads to a decreased quality of life, an increased risk of death, and higher medical expenses, ultimately placing a substantial burden on both patients and the healthcare system [2]. However, the pathogenesis of PND remains unclear, and there is a lack of specific intervention methods. Therefore, revealing its molecular mechanism holds great clinical importance and scientific significance [3].

An increasing number of studies confirm that postoperative neuroinflammation is a key driver of PND. As innate immune cells in the CNS, microglia serve as the primary regulators of neuroinflammation [4]. Surgical trauma triggers a systemic inflammatory response, which activates hippocampal microglia abnormally and promotes pro‐inflammatory factor release via multiple pathways including peripheral immune cell activation, blood–brain barrier disruption and inflammatory factor infiltration, thus initiating a neuroinflammatory vicious cycle [5]. Neuroinflammation causes neuronal damage, impaired synaptic plasticity, and inhibited neurogenesis, which together constitute the core pathological basis of PND [6]. However, the upstream signaling and epigenetic regulatory networks underlying microglial dysfunction remain poorly understood, and elucidating these networks is crucial for uncovering PND pathogenesis.

Recently, the intersection of metabolism and epigenetics provides a new perspective for understanding the role of neuroinflammation in PND. Microglial function is tightly regulated by metabolic patterns [7]; under postoperative stress, microglial energy metabolism shifts from oxidative phosphorylation (OXPHOS) to glycolysis, a key driver of their abnormal activation and M1 polarization, thereby promoting PND [8, 9]. Enhanced glycolysis leads to significant lactate accumulation, which mediates histone lactylation, a novel lactate‐driven epigenetic modification that promotes specific gene transcription by altering chromatin openness [10]. As a major histone lactylation site, H3K18 lactylation (H3K18la) plays a critical role in CNS diseases via neuroinflammation [11, 12]. In models of aging and Alzheimer's disease (AD), H3K18la directly promotes the transcriptional activation of nuclear factor kappa‐B (NF‐κB). This activation, in turn, leads to microglia senescence and the increased production of Interleukin (IL)‐6 and IL‐8, ultimately exacerbating cognitive dysfunction in mice [13]. In a mouse model of diabetes‐related cognitive impairment, H3K18la promotes the M1 polarization of microglia by up‐regulating the expression of Toll‐like receptor 4 (TLR4), which in turn aggravates neuroinflammation [14]. However, it remains unclear whether H3K18la plays a role in the development and progression of PND by regulating microglia function.

Lymphocyte activation gene 3 (Lag3) is a recently discovered immune checkpoint receptor expressed on the surface of various immune cells. It participates in the regulation of immune cell activity and the immune response [15, 16]. Previous studies have confirmed that Lag3 is mainly expressed on the surface of microglia in the CNS by single‐cell sequencing [17]. However, the role of Lag3 in CNS diseases remains to be fully validated. Some studies showed that Lag3 expression in brain microglia is significantly increased in chronically stressed mice. Additionally, intranasal infusion of Lag3 antibodies has demonstrated a significant antidepressant effect by inhibiting microglial activation via the ERK1/2‐BDNF signaling pathway [18]. In addition, in a mouse model of Parkinson's disease (PD), Lag3 knockout could improve behavioral deficits by inhibiting microglial activation [19]. The evidence suggests that Lag3 may play a crucial role in regulating microglia‐mediated neuroinflammation. However, the molecular mechanisms by which Lag3 influences microglia function and its specific role in PND require further elucidation.

In this study, we investigated the role of H3K18la in PND pathogenesis and its underlying mechanism. Our results demonstrated that surgery‐induced H3K18la accumulation in the hippocampus facilitates PND progression. Mechanistically, H3K18la upregulated Lag3 expression, thereby triggering microglial pyroptosis via the NOD‐like receptor protein 3 (NLRP3) pathway. This study firstly identifies the regulatory function of histone lactylation in microglial activity during PND, offering a novel theoretical basis and promising therapeutic target for PND clinical prevention and intervention.

## 2 Materials and Methods

### 2.1 Declaration

No written consent has been obtained from the patients as there is no patient‐identifiable data included.

### 2.2 Animals

Male C57BL/6 mice (8–12 weeks) were purchased from Harbin Baiqing Biotechnology Co. Ltd. Mice were housed 5 per cage under a 12 h light/dark cycle with free access to food and water. All experimental procedures were approved by the Institutional Animal Care and Use Committee (No. 2024‐DWSYLLCZ‐41).

### 2.3 Surgical Model and Treatment

Mice were anesthetized with sevoflurane and subjected to exploratory laparotomy as previously described [20]. After anesthesia and disinfection, a midline abdominal incision was performed. The liver, spleen, both kidneys, and intestines were examined for 10 min, and the incision was closed following 0.2% lidocaine local infiltration. Control mice received no anesthesia or surgery.

Mice were sacrificed at 12, 24, 48, and 72 h post‐surgery for tissue harvesting. For glycolysis inhibition, mice were randomized into Control (Con), Surgery (Sur), and Surgery+2‐DG (Sur + 2‐DG) groups. Sur + 2‐DG mice received 2‐DG (250 mg/kg; MedChemExpress, HY‐13966) [9] intraperitoneally once daily for 2 days preoperatively until 30 min before surgery. For Lag3 blockade, mice were divided into four groups (Con + Saline, Con + anti‐Lag3, Sur + Saline, Sur + anti‐Lag3); anti‐Lag3 monoclonal antibody (mAb) (100 μg; Biolegend, BLG‐125204) [21] or saline was injected intraperitoneally 30 min before surgery and on postoperative days 1 and 2. Behavioral tests were performed from postoperative day 3.

### 2.4 Cell Culture and Intervention

BV2 cells (National Infrastructure of Cell Line Resource, China) were cultured in high‐glucose DMEM with 10% FBS and 1% antibiotics at 37°C with 5% CO_2_.

First, cells were treated with 0, 1, 5, or 25 mM lactate (Aladdin, S108838) for 24 h for protein expression analysis. For inhibitor rescue experiments, BV2 cells were pretreated with selective p300 inhibitor A‐485 (0.5 μM; MedChemExpress, HY‐107455) for 12 h prior to incubation with 25 mM lactate for another 24 h. To mimic surgical stress, cells were pretreated with 25 mM lactate or 10 mM 2‐DG for 3 h, then stimulated with LPS (1 μg/mL; Beyotime, S1732) and IFN‐γ (50 ng/mL; Thermo Fisher, 315‐05) for 24 h. Previous studies have demonstrated that IFN‐γ significantly induces the expression of Lag3 in microglial cells [22].

### 2.5 Cell Transfection

Transient transfection was performed with Lipofectamine 8000 (Beyotime, C0533) to introduce Lag3‐specific siRNA (Santa Cruz Biotechnology, sc‐44548) or control siRNA (Santa Cruz Biotechnology, sc‐44230); 72 h post‐transfection, cells were treated with LPS and IFN‐γ for 24 h. For NLRP3 rescue experiments, transfected BV2 cells were sequentially treated with 5 μM Nigericin (MedChemExpress, HY‐127019) for 12 h, followed by combined stimulation with LPS and IFN‐γ for 24 h.

### 2.6 Behavioral Tests

All mice were randomly assigned to groups via random numbering, and their testing order was randomized to reduce order effects. All behavioral assessments, video tracking, and analyses were conducted by investigators who remained blinded to group allocation and treatments.

### 2.7 Open Field Test (OFT)

The OFT was conducted in an arena that was partitioned into a 3 × 3 grid of equal squares, where the central square was identified as the central area. Following a 300 s exploration session for each animal, the total distance traversed was automatically quantified by the SuperMaze video‐tracking system (XinRuan, Shanghai, China).

### 2.8 Novel Object Recognition (NOR)

Mice were habituated to an empty open field on day 1, trained with two identical objects on day 2 for 180 s, and tested with one familiar and one novel object on day 3. Exploration time was recorded, and memory performance was calculated as the percentage of time spent exploring the novel object.

### 2.9 Barnes Maze Test

Spatial learning and memory were assessed using the Barnes maze. Mice underwent acclimatization with aversive stimulation to locate the escape port within 180 s. Acquisition training was performed twice daily for 5 days; mice that failed to locate the port within 180 s were gently guided. After a 3‐day interval, a probe trial was conducted on day 9 with the escape port removed. Latency and distance to the target hole were recorded and analyzed with the SuperMaze video‐tracking system.

### 2.10 Measurement of Lactate Levels

Lactate levels in tissues, serum, or cells were quantified with CheKine micro‐Lactate Assay Kit (Abbkine, KTB1100).

### 2.11 Lactate Dehydrogenase (LDH) Assay

Cell culture supernatant was collected, and LDH release was determined using LDH assay kit (Nanjing Jiancheng Bioengineering Institute, A020‐2‐1) according to the manufacturer's instructions.

### 2.12 CUT&Tag and Data Processing

Fresh hippocampal tissues were harvested 24 h after surgery or from the control mice, snap‐frozen and submitted to Jiayin Biotechnology Ltd. (Shanghai, China). Raw FASTQ data were adapter‐trimmed and filtered using Perl scripts. Clean reads were aligned to the reference genome by BWA (v0.7.13‐r1126). Peak calling was performed with MACS2 (v2.1.2, *q* < 0.05). Peaks were annotated by BEDTools (v2.17.0), and de novo motifs were analyzed using HOMER (v4.11). GO and KEGG enrichment analyses were conducted via Fisher's exact test with FDR correction. Peak visualization was carried out using IGV v2.14.1.

### 2.13 Chromatin Immunoprecipitation (ChIP)‐qPCR

ChIP was conducted with the Magna ChIP A/G Kit (Magna0017, Magna, Canada) following the manufacturer's protocol. Briefly, cells were crosslinked with formaldehyde, neutralized with glycine, washed, and harvested. Cell lysis, chromatin sonication, and immunoprecipitation were completed as instructed. Chromatin was incubated with anti‐H3K18la antibody, and IgG served as the negative control. The enriched DNA was purified and subjected to qPCR with the following primers: Forward primer Sequence: TGGTGGTTGCTTTCTTTCGC; Reverse primer Sequence: CCACGTAAGCTTGGTGACCT.

### 2.14 RNA‐Seq and Data Processing

Hippocampal tissues were collected at 24 h postoperatively from 2‐DG‐treated and surgical mice, snap‐frozen and sent to Jiayin Biotechnology (Shanghai). BV2 cells transfected with si‐Lag3 or si‐NC were stimulated with LPS and IFN‐γ for 24 h, lysed in Trizol, snap‐frozen, and sent to APPLIED PROTEIN TECHNOLOGY (Shanghai). RNA‐seq of mouse hippocampus were sequenced on Illumina NovaSeq 6000; BV2 cell RNA‐seq was performed on the DNBSEQ (paired‐end 150 bp). Raw reads were processed using FastQC (v0.11.9), Trimmomatic (v0.39), STAR (v2.7.10a) and SAMtools (v1.16.1), with genome alignment against mm10. DEGs were identified by DESeq2 (|log_2_FC| > 1, *p* < 0.05), and GO/KEGG enrichment was analyzed via Dplyr (*p* < 0.05).

### 2.15 Nucleoprotein Extraction

Nuclear proteins were extracted using a commercial kit (Beyotime, China) according to the manufacturer's instructions.

### 2.16 Western Blotting (WB)

Total protein was extracted using RIPA buffer, and protein concentration was quantified with a BCA kit. Equal protein loads were separated by SDS‐PAGE, transferred to PVDF membranes, and blocked with 5% skim milk. Membranes were incubated overnight at 4°C with primary antibodies: Pan‐Kla (1:1000; PTM BIO, 1401RM), H3K18la (1:1000; PTM BIO, 1406RM), Lag3 (1:5000; Proteintech, 80867‐1‐RR), NLRP3 (1:1000; CST, 15101T), Caspase‐1 (1:50,000; HUABIO, ET1608‐69), Cleaved Caspase‐1 (1:1000; Wanleibio, WL03450), GSDMD‐N (1:1000; HUABIO, HA721144), IL‐1β (1:1000; Wanleibio, WL00891), IL‐18 (1:1000; Wanleibio, WL01127), Phospho‐NF‐κB (1:5000; Proteintech, 82335‐1‐RR), histone H3 (1:1000; Beyotime, AF0009), GAPDH (1:10,000; ABclonal, AC054), and β‐actin (1:10,000; ABclonal, AC026). Subsequently, membranes were incubated with HRP‐conjugated secondary antibodies (1:5000–1:10,000; ABclonal) for 60 min. Protein bands were visualized using an enhanced chemiluminescence reagent and quantified with ImageJ software.

### 2.17 Immunofluorescence (IF)

After anesthesia, mice were transcardially perfused with cold saline and 4% paraformaldehyde (PFA). Mouse brains were fixed, cryoprotected, embedded, and sectioned at 15 μm. BV2 cells were fixed in 4% PFA. Samples were blocked with 1% BSA containing 0.3% Triton X‐100 for 1.5 h, incubated with primary antibodies including Iba‐1 (1:200, Wako 011‐27991), H3K18la (1:100; PTM BIO, 1406RM), Lag3 (1:200; Proteintech, 80867‐1‐RR), Pan‐Kla (1:100; PTM BIO, 1401RM), Caspase‐1 (1:100; HUABIO, ET1608‐69), and GSDMD‐N (1:100; HUABIO, HA721144) overnight at 4°C, then treated with fluorescent secondary antibodies and DAPI. Imaging was performed with an Olympus confocal microscope.

### 2.18 Statistical Analysis

Data are presented as mean ± SD. All data were tested for normality and analyzed with SPSS 25.0 (version 25.0; IBM, USA). Comparisons between two groups used unpaired Student's *t*‐test; multiple groups were analyzed by one‐way ANOVA with Tukey's post hoc test. *p* < 0.05 was considered statistically significant.

## 3 Results

### 3.1 Surgery Elevates Hippocampal Lactate and Upregulates Histone Pan‐Kla and H3K18la

**FIGURE 1:**
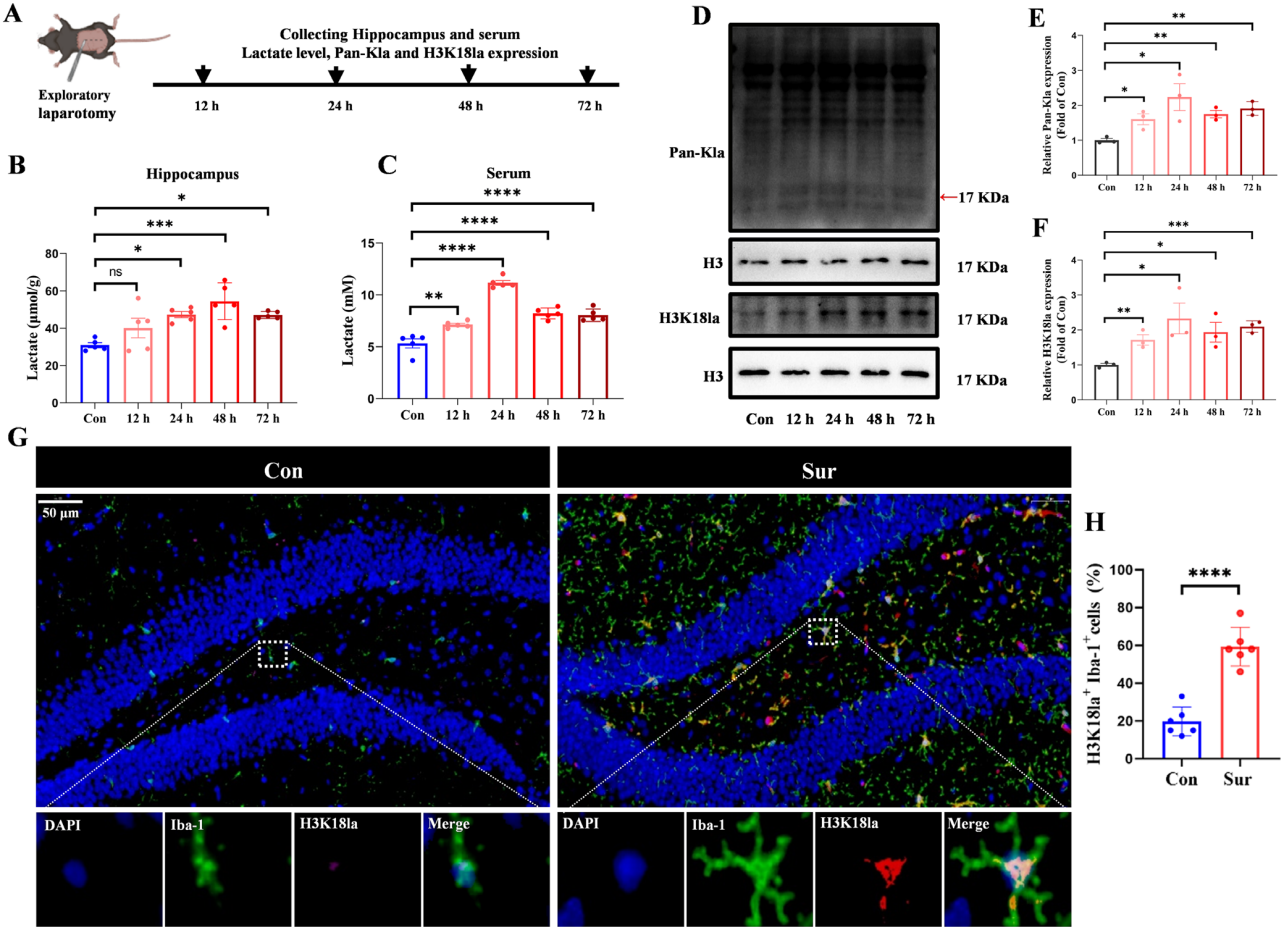
Upregulation of hippocampal lactate, histone Pan‐Kla, and H3K18la following surgery. (A) Collect the mouse hippocampal tissue and serum samples at 12, 24, 48 and 72 h after laparotomy exploration. (B, C) Lactate levels in the hippocampus and serum of the postoperative mice (*n* = 5). (D) Hippocampal levels of histone Pan‐Kla and H3K18la after surgery were assessed by immunoblotting, with histone H3 used for normalization. (E, F) Quantitation of Pan‐Kla and H3K18la gray value (*n* = 3). (G) Representative IF images of H3K18la in hippocampal Iba1^+^ cells. (H) Proportions of H3K18la^+^/Iba1^+^cells (*n* = 6). Data are mean ± SD. Statistical significance was assessed by unpaired 2‐tailed Student *t*‐test compared with Con group. *p < 0.05, **p < 0.01, ***p < 0.001, and ****p < 0.0001.

We first measured lactate levels in the hippocampus and serum of mice at various postoperative time points (Figure 1A). Hippocampal lactate levels significantly increased at 24 h post‐surgery and remained elevated until 72 h (Figure 1B), while serum lactate levels were significantly higher than those in the control group from 12 to 72 h post‐surgery (Figure 1C). Additionally, hippocampal Pan‐Kla and H3K18la levels were significantly elevated from 12 to 72 h post‐surgery (Figure 1D–F). IF results showed a significant increase in the percentage of H3K18la^+^/Iba‐1^+^ microglia in the dentate gyrus (DG) area of the hippocampus after surgery (Figure 1G,H). These results suggest that elevated H3K18la in hippocampal microglia post‐surgery may be involved in the development of PND.

### 3.2 Intervention With Glycolysis Inhibitor Alleviates Cognitive Impairment in Mice After Surgery

To investigate the role of histone lactylation in PND pathogenesis, mice received intraperitoneal 2‐DG, and behavioral tests were performed from postoperative day 3 (Figure S1A). The OFT showed no significant differences in total travel distance, central area duration and central entries across all groups (Figure S1B–D). These comparable OFT results exclude locomotor activity as a confounding factor for the subsequent NOR and Barnes maze tests. In NOR, the Sur group showed reduced novel object exploration compared with the Con group, which was reversed by 2‐DG (Figure S1E,F). Barnes maze showed that the Sur group had longer target distance on training day 1 and longer escape latency on days 2, 4, 5, which were improved by 2‐DG (Figure S1G,H). In the probe trial, 2‐DG ameliorated the prolonged distance and latency in the Sur group (Figure S1I–K). No significant difference in average speed was observed among groups (Figure S1L).

### 3.3 H3K18la Facilitates the Transcription of the Immune Checkpoint Gene Lag3

**FIGURE 2:**
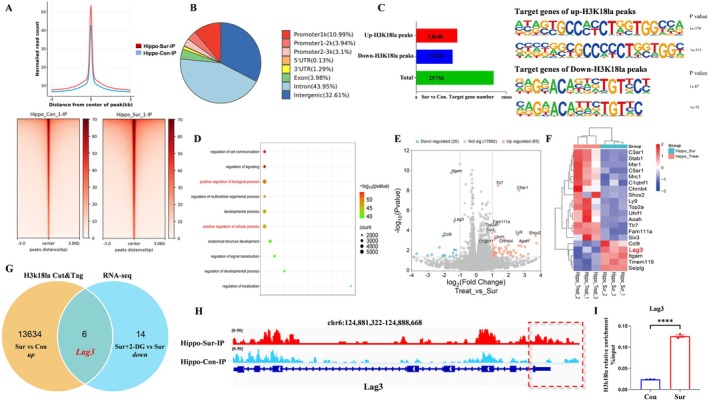
The target genes regulated by H3K18la were identified by CUT&Tag assay combined with RNA‐seq. (A) Average plot and heat map displaying the binding density of H3K18la with different H3K18la binding peaks in hippocampus of the Sur group and Con group. (B) Genome‐wide distribution of the upregulated H3K18la‐binding peaks in mice after surgery. (C) Bar graph illustrating the number of target genes associated with upregulated and downregulated H3K18la peaks, and the top 2 enriched de novo motifs of target genes. (D) GO analysis of candidate target genes associated with upregulated H3K18la peaks. (E) Volcano plot of differentially expressed genes in RNA‐seq (*n* = 3). (F) Heat map showing TOP 20 differential genes after 2‐DG treatment in RNA‐seq (*n* = 3). (G) Venn diagram showing the intersection of H3K18la up‐regulated target genes and 2‐DG down‐regulated target genes. (H) Genome browser tracks show H3K18la binding at representative target gene loci. Peaks specifically located at the Lag3 promoters are identified by red rectangles. (I) H3K18la occupancy analysis by ChIP‐qPCR (*n* = 3). Data are mean ± SD. Statistical significance was assessed by unpaired 2‐tailed Student *t*‐test compared with Con group. ****p < 0.0001.

**FIGURE 3:**
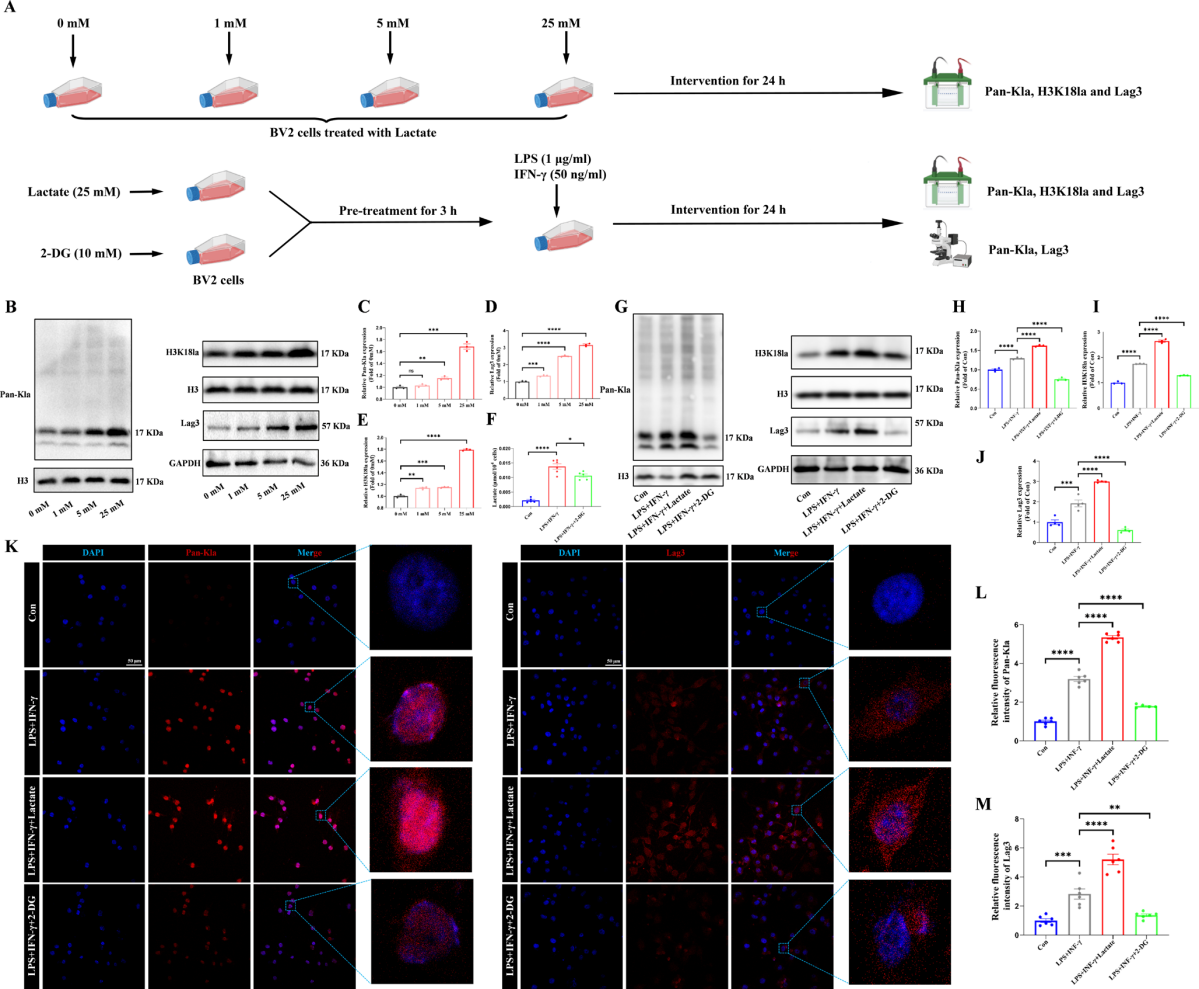
H3K18la promotes Lag3 expression in microglial cells induced by either lactate or LPS and IFN‐γ. (A) BV2 cells were first treated with 0‐, 1‐, 5‐, and 25‐mM lactate for 24 h. Subsequently, cells pretreated with 25 mM lactate or 10 mM 2‐DG for 3 h were stimulated with LPS and IFN‐γ for 24 h. (B) Representative immunoblotting of Pan‐Kla, H3K18la and Lag3 in BV2 cells intervention with lactate, with histone H3 or GAPDH used for normalization. (C‐E) Quantitation of Pan‐Kla, H3K18la and Lag3 gray value (*n* = 3). (F) The lactate levels of BV2 cells activated by LPS and IFN‐γ (*n* = 5–6). (G) Representative immunoblotting of Pan‐Kla, H3K18la and Lag3 in BV2 cells activated by LPS and IFN‐γ, with histone H3 or GAPDH used for normalization. (H–J) Quantitation of Pan‐Kla, H3K18la and Lag3 gray value (*n* = 3–4). (K) Representative fluorescence images of Pan‐Kla or Lag3 in BV2 cells. (L, M) The relative fluorescence intensities of Pan and Lag3 in BV2 cells (*n* = 6). The images include scale indicators of 50 μm for size reference. The data are presented as the mean ± SD, A–D were assessed by unpaired 2‐tailed Student *t*‐test compared with Con group. The other results were assessed by one‐way ANOVA followed by Tukey's post hoc test. *p < 0.05, **p < 0.01, ***p < 0.001, ****p < 0.0001.

CUT&Tag results showed significantly elevated H3K18la enrichment in the hippocampus of the Sur group versus controls (Figure 2A). In total, 25,756 significantly upregulated H3K18la binding peaks and 19,289 downregulated peaks were identified in the Sur group. Among these 25,756 upregulated H3K18la peaks, 10.99% were annotated to proximal promoter regions (≤ 1 kb), while 3.94% were located in distal promoter regions (1–2 kb) (Figure 2B). Genomic annotation revealed that upregulated peaks were mapped to 13,640 target genes, while downregulated peaks corresponded to 12,116 target genes. De novo motif analysis identified distinct binding sequence characteristics between target genes associated with upregulated and downregulated H3K18la peaks (Figure 2C). GO enrichment was performed with an FDR < 0.05. GO enrichment analysis showed that positive regulation of biological processes (FDR = 1.60 × 10^−122^) and positive regulation of cellular processes (FDR = 5.74 × 10^−126^) were included among the top 10 enriched GO terms for genes associated with elevated H3K18la peaks following surgical intervention (Figure 3D), demonstrating that elevated H3K18la modification is critically involved in the development of microglial dysfunction in PND.

RNA‐seq identified 20 significantly downregulated genes post 2‐DG treatment (|log_2_FC| > 1, *p* < 0.05) (Figure 2E,F), and integrative analysis of CUT&Tag and RNA‐seq data yielded six overlapping genes including *Asmt, Ccl9, Lag3, Gnb3, H2‐Oa* and *Selplg* (Figure 2G). Among the six overlapping genes, Lag3 displayed the lowest *p* value, reflecting the most significant transcriptional change. Furthermore, Lag3 is a key immune checkpoint receptor that modulates immune activity and inflammatory responses [15, 16]. In CNS, single‐cell sequencing has verified that Lag3 is predominantly expressed in microglia, but its function in microglial regulation and neuroinflammation remains undefined. Thus, Lag3 was selected as the downstream target gene regulated by H3K18la. Specifically, the peaks identified candidate genomic loci at the Lag3 gene (gene ID: 16768, location: chr6: 124,881,322–124,888,668), showing that the abundance of H3K18la binding on the promoter of the Lag3 gene in the hippocampus of postoperative mice was significantly elevated compared to control mice (Figure 3H). Mouse Lag3 is transcribed from the minus strand of chromosome 6 in the reverse direction. Its promoter and TSS are positioned at the higher coordinate end of the gene locus. The red‐boxed H3K18la peak in Figure 2H is enriched within this region, which is consistent with the reverse transcriptional orientation of Lag3 (Figure 2H). To validate the CUT&Tag and RNA‐seq data in the hippocampus of mice after surgery, we further conducted ChIP‐qPCR analysis to confirm the increased H3K18la enrichment in the promoter regions of Lag3 (Figure 2I).

### 3.4 Inhibition of Histone H3K18la After Surgery Inhibits the Expression of Lag3 in Mouse Hippocampal Microglia

After glycolysis inhibition, WB results showed that hippocampal Lag3 protein levels increased markedly from 12 to 72 h after surgery, with a peak at 24 h (Figure S2A–C). 2‐DG intervention effectively reduced hippocampal Pan‐Kla and H3K18la expression in postoperative mice, accompanied by obvious downregulation of Lag3 expression (Figure S2D–H). Immunofluorescence further confirmed that the number of Lag3^+^/Iba1^+^ microglia was markedly elevated after surgery, and this increase was obviously reversed by 2‐DG treatment (Figure S2I,J). Collectively, these in vivo findings demonstrate that surgery‐induced H3K18la upregulation promotes Lag3 expression in hippocampal microglia.

### 3.5 In Vitro Experiments Verified That H3K18la Promotes the Expression of Lag3 in Microglial Cells Induced by Lactate or LPS and IFN‐γ

The in vitro experimental protocol illustrated in Figure 3A. Lactate dose‐dependently elevated Pan‐Kla and H3K18la levels, along with Lag3 expression in BV2 cells (Figure 3B–E). LPS and IFN‐γ stimulation increased lactate accumulation, whereas 2‐DG intervention reduced lactate production (Figure 3F). Compared with the Con group, LPS and IFN‐γ upregulated Pan‐Kla and H3K18la; lactate further enhanced their expression, while 2‐DG exerted an inhibitory effect (Figure 3G–I). Importantly, Lag3 expression was significantly higher in the LPS + IFN‐γ group than in the Con group, and lactate addition further increased Lag3 levels, whereas 2‐DG inhibited its expression (Figure 3G,J). IF verified elevated nuclear Pan‐Kla and membrane Lag3 fluorescence in LPS and IFN‐γ activated BV2 cells, which was attenuated by 2‐DG (Figure 3K–M). In addition, since 2‐DG globally suppresses glycolysis and disrupts multiple lactate‐independent metabolic pathways, we treated BV2 cells with A‐485, a selective inhibitor of the histone lactylation catalytic enzyme p300. We found that A‐485 treatment significantly abolished lactate‐induced upregulation of H3K18la and Lag3 expression in BV2 cells (Figure S3A–C). Collectively, these findings suggest that elevated lactate upregulates Lag3 expression by enhancing H3K18la during microglial overactivation.

### 3.6 Administration of the Anti‐Lag3 Monoclonal Antibody Ameliorated the Cognitive Decline Following Surgery in Mice

**FIGURE 4:**
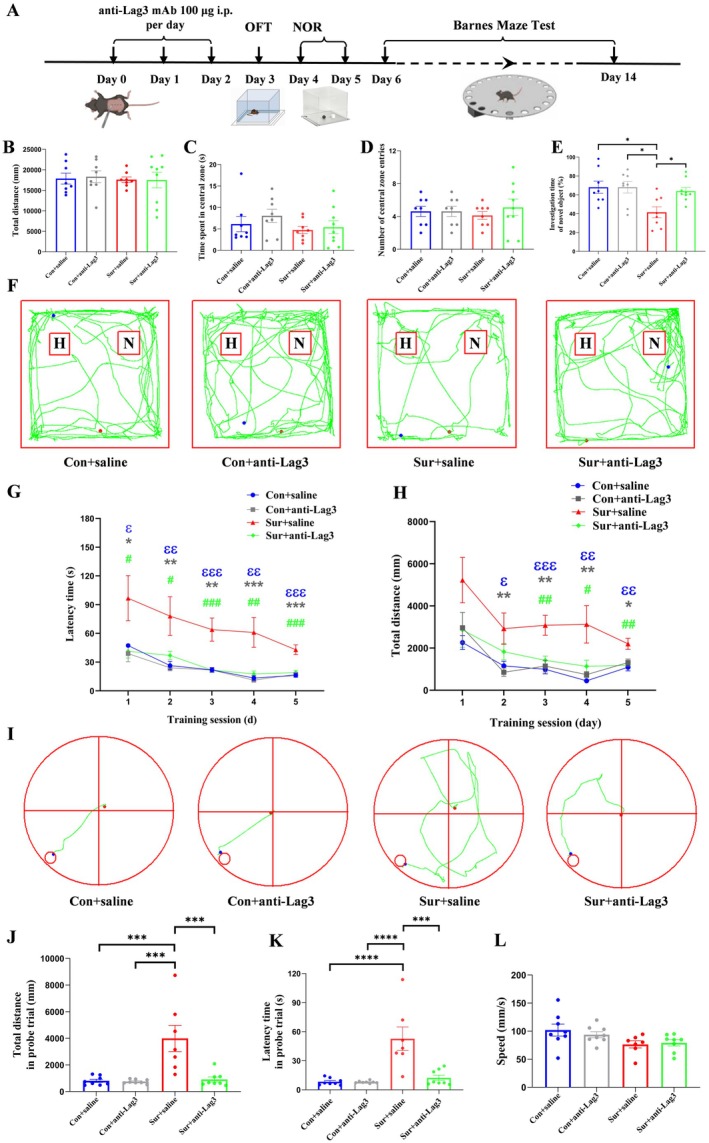
Treatment with the anti‐Lag3 mAb alleviates the cognitive decline induced by surgery in mice. (A) Timeline of anti‐Lag3 mAb intervention strategy and behavioral assessment. (B) Total distance in the OFT (*n* = 8–9). (C) Time spent in central zone (*n* = 8–9). (D) Numbers of entering the central zone (*n* = 8–9). (E) Percentage of investigation time of novel object in the NOR (*n* = 8–9). (F) Representative trajectories in the NOR. (G, H) Total distance and latency in Barnes maze acquisition training (*n* = 7–8). (I) Typical trajectories within the probe trial procedure. (J) Total distance in probe trial latency (*n* = 7–8). (K) Latency time in probe trial (*n* = 7–8). (L) Mean velocity of movement (*n* = 7–8). The data are presented as the mean ± SD; one‐way ANOVA was employed followed by Tukey's post hoc test. *p < 0.05, **p < 0.01, ***p < 0.001, ****p < 0.0001; *p* < 0.05, *p* < 0.01, *p* < 0.001, Sur + saline group versus Con+saline group; ^#^ *p* < 0.05, ^##^ *p* < 0.01, ^###^ *p* < 0.001, Sur + anti‐Lag3 group versus Sur + saline group.

As illustrated in Figure 4, OFT results revealed no significant differences in total travel distance, central area residence time, or entry count among all groups (Figure 4A–D). In the NOR test, the Sur + saline group exhibited significantly reduced novel object exploration time compared with the Con+saline group, whereas the Sur + anti‐Lag3 group showed a significant increase relative to the Sur + saline group (Figure 4E,F). Barnes maze assessments demonstrated that the Sur + anti‐Lag3 group had significantly shorter latency and reduced total travel distance compared with the Sur + saline group during both training and testing phases (Figure 4G–K). Notably, no significant differences in locomotor speed were observed among groups (Figure 4L), indicating that anti‐Lag3 mAb treatment mitigates postoperative spatial memory impairment in mice.

### 3.7 RNA‐Seq in BV2 Cells Revealed That Lag3 Promotes Microglial Pyroptosis via the NLRP3 Pathway

**FIGURE 5:**
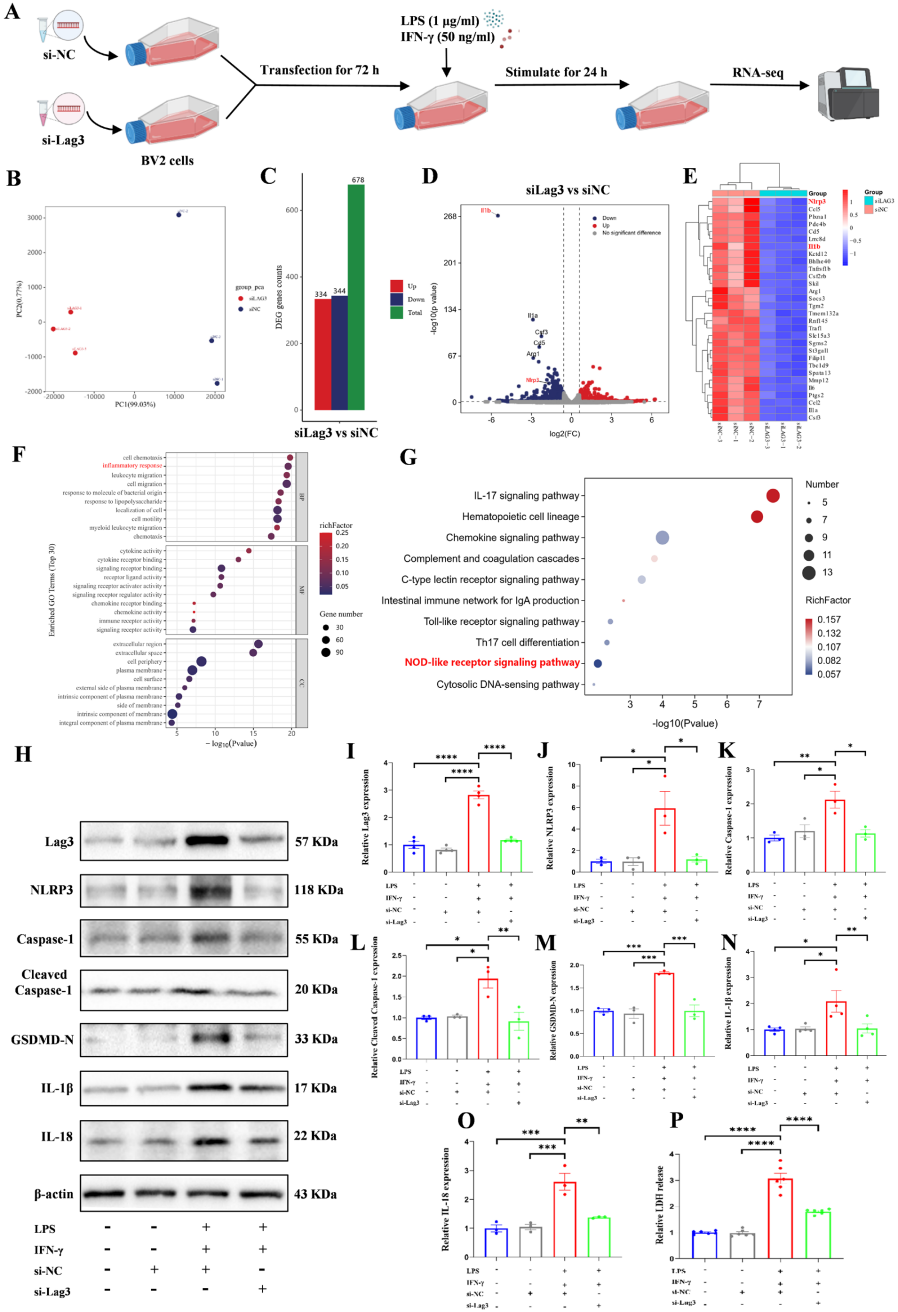
Knockdown of Lag3 in BV2 cells inhibited the expression of pyroptosis‐related genes. (A) After transfecting BV2 cells with si‐NC or si‐Lag3 for 72 h, LPS and IFN‐γ were applied for 24 h, and then total RNA was collected for RNA‐seq. (B) Principal component analysis (PCA) (*n* = 3). (C) Bar graph indicating the number of up‐regulated and downregulated genes after transfection with si‐Lag3. (D) Volcano plot of differentially expressed genes in RNA‐seq. (E) Heat map showing the TOP 30 downregulated genes after transfection with si‐Lag3 sorted in descending order based on *p*‐value. (F) GO analysis of downregulated genes after transfection with si‐Lag3. (G) Bubble diagram showing KEGG pathway enriched with downregulated genes. (H) Presentation of WB results showing the expression of Lag3, NLRP3, Caspase‐1, Cleaved Caspase‐1, GSDMD‐N, IL‐1β and IL‐18, with β‐Actin serving as the reference for equal loading. (I–O) Quantitation of gray value (*n* = 3–4). (P) Relative LDH release measured in cell culture supernatants (*n* = 6). The data are presented as the mean ± SD, one‐way ANOVA was employed followed by Tukey's post hoc test. *p < 0.05, **p < 0.01, ***p < 0.001, ****p < 0.0001.

To elucidate the mechanism by which microglial Lag3 upregulation promotes PND, we performed RNA‐seq in LPS/IFN‐γ‐stimulated BV2 cells after Lag3 knockdown (Figure 5A). Principal component analysis (PCA) showed distinct transcriptomic profiles between si‐NC and si‐Lag3 groups (Figure 5B). Lag3 knockdown altered 678 genes (334 upregulated, 344 downregulated, Figure 5C), with significant downregulation of IL‐1β (log_2_FC = −5.51, FDR < 0.0001) and NLRP3 (log_2_FC = −1.70, FDR < 0.0001) (Figure 5D,E). GO/KEGG analyses enriched these downregulated genes in inflammatory response and NOD‐like receptor signaling pathways (Figure 5F,G). NLRP3 inflammasome activation is a key driver of microglial pyroptosis and neuroinflammation [23, 24]. We thus hypothesized that Lag3 upregulation may trigger microglial pyroptosis via NLRP3 signaling.

WB confirmed that Lag3 knockdown suppressed LPS/IFN‐γ‐induced expression of NLRP3, Caspase‐1, Cleaved Caspase‐1, GSDMD‐N, IL‐1β, and IL‐18 (Figure 5H–O). In addition, we measured supernatant LDH release in BV2 cells and found that LPS/IFN‐γ treatment elevated LDH efflux, which was significantly inhibited by Lag3 knockdown (Figure 5P). These functional data further confirm that Lag3 upregulation drives microglial pyroptosis. To further validate the epistatic relationship between Lag3 and NLRP3, we treated Lag3‐knockdown BV2 cells with the NLRP3 agonist nigericin. p‐NF‐κB levels were markedly elevated in the LPS + IFN‐γ + si‐NC group relative to the Con group, whereas Lag3 knockdown attenuated p‐NF‐κB expression. Nigericin treatment did not alter p‐NF‐κB levels in Lag3‐knockdown cells (Figure S4A,B), but significantly restored the expression of pyroptotic markers including Caspase‐1, GSDMD‐N, and IL‐1β compared with the LPS + IFN‐γ + si‐Lag3 group (Figure S4A,C–E). As NF‐κB signaling acts as an essential upstream trigger for NLRP3 inflammasome priming, these findings demonstrate that elevated microglial Lag3 drives NLRP3‐dependent microglial pyroptosis via NF‐κB pathway activation.

### 3.8 Inhibition of Glycolysis Alleviates NLRP3 Pathway‐Mediated Microglial Pyroptosis in the Hippocampus of Mice After Surgery

To determine whether H3K18la induces microglial pyroptosis in the hippocampus of postoperative mice, IF co‐localization of key pyroptosis proteins, Caspase‐1 and GSDMD‐N with Iba‐1 showed increased Caspase‐1^+^/Iba‐1^+^ and GSDMD‐N^+^/Iba‐1^+^ cells in the Sur group, which were reduced by 2‐DG (Figure S5A–D). WB demonstrated elevated NLRP3, Caspase‐1, GSDMD‐N, and IL‐1β in Sur group hippocampi, with significant reduction in Sur + 2‐DG mice (Figure S5E–I). These results suggest that the increase in H3K18la expression may play a crucial role in inducing pyroptosis of hippocampal microglia in mice following surgery.

### 3.9 Treatment With the Anti‐Lag3 mAb Inhibits Microglial Pyroptosis in the Hippocampus of Mice After Surgery

**FIGURE 6:**
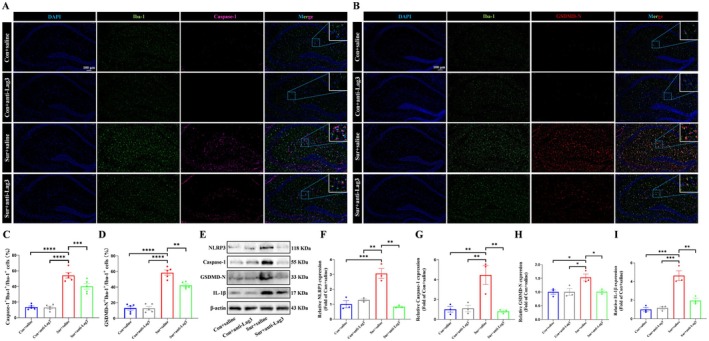
Anti‐Lag3 mAb treatment inhibits surgery‐induced microglial pyroptosis in the mouse hippocampus. (A, B) Representative IF images of Caspase‐1 and GSDMD‐N in hippocampal Iba1^+^ cells. (C, D) Proportions of Caspase1^+^/Iba‐1^+^ and GSDMD‐N^+^/Iba‐1^+^ cells (*n* = 3). (E) Representative immunoblots of NLRP3, Caspase‐1, GSDMD‐N and IL‐1β. (F–I) Quantified gray value of NLRP3, Caspase‐1, GSDMD‐N and IL‐1β (*n* = 3). The data are presented as the mean ± SD, one‐way ANOVA was employed followed by Tukey's post hoc test. *p < 0.05, **p < 0.01, ***p < 0.001, and ****p < 0.0001.

Following the administration of anti‐Lag3 mAb in vivo post‐surgery, IF results indicated a significant reduction in the number of Caspase‐1^+^/Iba‐1^+^ and GSDMD‐N^+^/Iba‐1^+^ cells in the mouse hippocampus (Figure 6A–D). Concurrently, WB analysis of pyroptosis‐related proteins demonstrated that, compared to the Sur + saline group, the expression of NLRP3, Caspase‐1, GSDMD‐N, and IL‐1β proteins in the hippocampus of mice in the Sur + anti‐Lag3 group was significantly decreased (Figure 6E–I). These findings further support the notion that upregulation of Lag3 expression in microglia after surgery can induce pyroptosis through the NLRP3 pathway, ultimately promoting PND.

## 4 Discussion

Recent studies have shown that lactate acts as both a byproduct and signaling molecule, activating gene transcription via histone lactylation [10]. However, the role of histone lactylation in PND remains unclear. The present study demonstrates surgical trauma‐induced hippocampal H3K18la elevation promotes PND. Epigenetically, H3K18la upregulates Lag3 and activates the downstream NLRP3 pathway, exacerbating neuroinflammation and neurological deficits in PND mice. Histone lactylation, a novel epigenetic modification, regulates gene transcription to maintain physiology and initiate pathology [25, 26]. Emerging evidence indicates that histone lactylation participates in the development of AD, cerebral ischemia, PD and other CNS diseases by regulating microglia function [27, 28, 29]. This study is the first to show postoperative mice have increased hippocampal lactate and H3K18la; inhibiting glycolysis to reduce H3K18la can suppress microglial pyroptosis in vivo/in vitro and alleviate cognitive impairment of PND mice.

H3K18la, a key histone lactylation site, plays a crucial role in microglia‐mediated neuroinflammation. For instance, in AD models, increased H3K18la in the hippocampus was shown to directly activate the microglial NF‐κB pathway, exacerbating brain aging by elevating the production of IL‐6 and IL‐8 [13]. Yang et al. showed that enhanced H3K18la promotes microglial M1 polarization via TLR4 signaling, aggravating diabetic cognitive impairment [14]. These studies support our finding that H3K18la promotes neuroinflammation by regulating microglial function, implying it is a key epigenetic mechanism in PND. However, a recent study revealed that H3K18la can also alleviate neuroinflammation and neural damage in ischemic stroke by upregulating microglial expression of plxnb2 [29]. This discrepancy may result from H3K18la regulating different microglial target genes via distinct molecular mechanisms in different CNS diseases. Thus, further investigation into the specific target genes and signaling pathways of H3K18la in PND is essential.

In our study, CUT&Tag combined with RNA‐seq revealed that H3K18la promotes microglial immune checkpoint Lag3 gene transcription. We further confirmed that lactate‐induced H3K18la elevation upregulates Lag3 expression in postoperative mouse hippocampal microglia and in BV2 cells treated with LPS and IFN‐γ. Importantly, anti‐Lag3 mAb treatment significantly alleviated cognitive impairment in PND model mice, providing preliminary evidence that microglial Lag3 upregulation contributes to PND development. Lag3 is a critical checkpoint for peripheral immune cells [30]. Under pathological conditions such as infection and tumor, the expression of Lag3 in immune cells including T cells and NK cells is increased, which further regulates the activity of immune cells [31, 32]. In the CNS, previous studies showed that Lag3 expression in microglia is significantly elevated in the brains of mice with depression models. Additionally, the blockade of the Lag3 checkpoint can notably alleviate depressive‐like symptoms [18, 21]. Another study discovered that the absence of Lag3 significantly decreased microglia activation and improved behavioral deficits in a mouse model of PD [19]. This evidence suggests that the microglial Lag3 may play a critical role in mediating cognitive dysfunction, aligning with our results. Typically, Lag3 binds to its ligands, such as MHC‐II, to transmit inhibitory signals, inhibiting T cell activation and exerting immune tolerance [31]. In contrast, our study shows that Lag3 promotes hippocampal microglial pyroptosis and exacerbates neuroinflammation post‐surgery, highlighting cell type and microenvironment‐dependent functional differences of a single molecule. In PND, surgical trauma‐induced systemic inflammation, metabolic stress, and gut microbiota imbalance may facilitate pro‐inflammatory non‐classical ligand binding to microglial Lag3. A previous study showed that misfolded α‐synuclein preformed fibrils can act as a Lag3 ligand, promoting endocytosis and neuronal death [33], partially supporting this speculation, but further exploration is needed in future studies.

Multiple studies confirm that NLRP3 pathway‐mediated microglial pyroptosis contributes significantly to PND [34, 35]. Microglial surface receptors initiate intracellular pro‐inflammatory pathways upon detecting harmful stimuli, such as damage‐associated molecular patterns (DAMPs). The activation of these receptors can lead to NLRP3‐mediated pyroptosis. For instance, classical pattern recognition receptors, including TLRs, can activate the NF‐κB and mitogen‐activated protein kinase (MAPK) pathways, subsequently triggering NLRP3‐mediated pyroptosis [36, 37], while P2X7 receptor activation can induce K^+^ outflow to promote NLRP3 oligomerization and pyroptosis [38, 39]. However, the role of microglial Lag3 in regulating pyroptosis remains unreported. In this study, RNA‐seq showed siRNA‐mediated Lag3 knockdown significantly downregulated IL‐1β and NLRP3 expression in LPS/IFN‐γ‐treated BV2 cells, with these genes enriched in the NOD‐like receptor signaling pathway. Further in vitro and in vivo experiments confirmed that Lag3 knockdown and anti‐Lag3 mAb treatment significantly inhibited microglial pyroptosis‐related key protein expression, indicating NLRP3‐mediated pyroptosis is a downstream effect of microglial Lag3 upregulation. Additionally, some evidence supports the Lag3‐NLRP3 regulatory relationship. A study in pancreatic cancer found that increased Lag3 expression in T cells significantly promoted NLRP3 expression [40]. More importantly, NF‐κB signaling serves as a vital upstream driver of NLRP3 inflammasome activation [41]. Zhao et al. illustrated that Lag3 triggers NF‐κB pathway activation via upregulating chitinase‐3‐like protein 1 in glioma cells [42], an event that likely enhances the transcriptional activity of NLRP3. Consistent with these published results, we detected robustly elevated phosphorylated NF‐κB levels in BV2 cells induced by LPS and IFN‐γ. Knockdown of Lag3 reversed this increase in phosphorylated NF‐κB, whereas treatment with an NLRP3 agonist exerted no obvious effect on NF‐κB phosphorylation. These results imply that microglial Lag3 upregulation may activate the NLRP3 inflammasome through the NF‐κB signaling cascade, thereby driving microglial pyroptosis. Nevertheless, the precise mechanism by which microglial Lag3 upregulation activates the NLRP3 pathway is complex and requires further investigation. However, our findings suggest that microglial Lag3 could serve as a potential new target for treating PND.

This study still has some limitations. First, non‐histone protein lactylation, which profoundly modulates microglial function, was not explored. Future studies using mass spectrometry and site mutation assays are required to clarify lactylation‐mediated regulation of microglial functional proteins. Moreover, histone acetylation is known to participate in PND progression via regulating microglial gene expression [43, 44], and the interplay between histone lactylation and acetylation remains to be elucidated. Given the complexity of PND pathogenesis, bulk hippocampal lactate quantification cannot resolve cell‐specific lactate production, precluding confirmation that lactate accumulation derives exclusively from microglia. Accordingly, it remains elusive whether lactate accumulation and histone lactylation directly modulate neuronal and astrocytic biological functions to promote PND, findings that require further research for definitive validation. Lastly, our research did not set up a group of mice receiving only anesthesia. Prior works have demonstrated that conventional doses of anesthesia alone do not trigger neuroinflammation or cognitive deficits in mice [45, 46]. Nevertheless, adding this control group could further improve the comprehensiveness of our study. In addition, we only employed adult male mice in the present study, and future verification using aged and female animals is required to better validate the clinical translational value of the H3K18la‐Lag3‐NLRP3 axis.

## 5 Conclusion

In conclusion, we elucidate that surgery induced elevation of microglial H3K18la drives PND pathogenesis. H3K18la upregulates Lag3 to trigger NLRP3‐dependent microglial pyroptosis and neuroinflammation, while Lag3 blockade alleviates PND (Graphical abstract). Our study offers new insights into the role of microglia‐mediated neuroinflammation in PND and identifies a novel target for potential interventions in its treatment.

## Author Contributions

S.Q. and C.L. conceived and designed this study. C.L., X.G. and Y.Z. performed the experiments. L.Z., S.L., S.D. and Q.L. analyzed the data. C.L. and S.Q. wrote and edited the manuscript. All authors read and approved the final manuscript.

## Funding

This study was supported by National Natural Science Foundation of China (82271207); The Cultivation Project of the Joint Fund of Heilongjiang Province Natural Science Foundation (PL2024H144); Outstanding Young Project of the Fourth Affiliated Hospital of Harbin Medical University (HYDSYYXQN202306); The Young Talent Research Project for Awarding Challenges at the Fourth Affiliated Hospital of Harbin Medical University (KYJB2024‐05).

## Ethics Statement

All animal experiments were approved by the Animal Care and Use Committee of Harbin Medical University, and in accordance with NIH Guidelines for the Care and Use of Laboratory Animals (ID: 2024‐DWSYLLCZ‐41).

## Consent

The authors have nothing to report.

## Conflicts of Interest

The authors declare no conflicts of interest.

## Supporting information


**Figure S1:** Inhibition of glycolysis alleviates cognitive impairment induced by surgery in mice. (A) Timeline of 2‐DG intervention strategy and behavioral assessment. (B) Total distance in the OFT (*n* = 9). (C) Time spent in central zone (*n* = 9). (D) Numbers of entering the central zone (*n* = 9). (E) Percentage of investigation time of novel object in the NOR (*n* = 9). (F) After adaptation on the first day and exploration of two identical objects on the second day, a new object was replaced on the third day, and representative trajectories in the NOR were shown. (G, H) Total distance and latency in Barnes maze acquisition training (*n* = 9). (I) Typical trajectories within the probe trial procedure. (J) Total distance in probe trial latency (*n* = 9). (K) Latency time in probe trial (*n* = 9). (L) Mean velocity of movement (*n* = 9). The data are presented as the mean ± SD, one‐way ANOVA was employed followed by Tukey's post hoc test. *p < 0.05, **p < 0.01. ^#^ *p* < 0.05, ^##^ *p* < 0.01, Sur + 2‐DG group vs. Sur group.
**Figure S2:** Lag3 expression of microglia in mice hippocampus after surgery and after treatment with 2‐DG. (A) Mouse hippocampi were collected at 12 h, 24 h, 48 h, 72 h post‐surgery and after 2‐DG treatment, followed by WB and IF analysis of Lag3 expression in microglia. (B) Representative immunoblotting of Lag3 in mice hippocampus after surgery, with β‐actin used for normalization. (C) Quantitation of Lag3 gray value (*n* = 4). (D) Hippocampal levels of histone Pan‐Kla and H3K18la after treatment with 2‐DG, with histone H3 used for normalization. (E, F) Quantitation of Pan‐Kla and H3K18la gray value (*n* = 3). (G) Representative immunoblotting of Lag3 in mice hippocampus after treatment with 2‐DG, with β‐actin used for normalization. (H) Quantitation of Lag3 gray value (*n* = 3). (I) Representative fluorescence images of Lag3 in Iba‐1^+^cells in the hippocampus. (J) Lag3^+^/Iba‐1^+^ cell proportion (*n* = 3). The images include scale indicators of 50 μm for size reference. The data are presented as the mean ± SD, one‐way ANOVA was employed followed by Tukey's post hoc test. *p < 0.05, **p < 0.01, ***p < 0.001, ****p < 0.0001.
**Figure S3:** Inhibition of p300 by A‐485 abolishes lactate‐induced upregulation of H3K18la and Lag3 in BV2 cells. (A) Representative immunoblotting of H3K18la and Lag3 in BV2 cells, with histone H3 or β‐actin used for normalization. (B, C) Quantitation of H3K18la and Lag3 gray value (*n* = 3). The data are presented as the mean ± SD, one‐way ANOVA was employed followed by Tukey's post hoc test. *p < 0.05, **p < 0.01, ***p < 0.001.
**Figure S4:** NLRP3 agonist nigericin reverses Lag3 knockdown‐mediated inhibition of LPS/IFN‐γ‐induced pyroptosis in BV2 cells. (A) Representative immunoblotting of p‐NF‐κB, GSDMD‐N, Caspase‐1 and IL‐1β in BV2 cells, with β‐actin used for normalization. (B‐E) Quantitation of p‐NF‐κB, GSDMD‐N, Caspase‐1 and IL‐1β gray value (*n* = 3). The data are presented as the mean ± SD, one‐way ANOVA was employed followed by Tukey's post hoc test. *p < 0.05, **p < 0.01, ***p < 0.001, ****p < 0.0001.
**Figure S5:** Inhibition of glycolysis reduces surgery‐induced microglial pyroptosis in the mouse hippocampus. (A, B) Representative fluorescence images of Caspase‐1 and GSDMD‐N expressions in Iba‐1^+^ cells in the hippocampus. (C) Caspase‐1^+^/Iba‐1^+^ cell proportion (*n* = 3). (D) GSDMD‐N^+^/Iba‐1^+^ cell proportion (*n* = 3). (E) Representative immunoblotting of NLRP3, Caspase‐1, GSDMD‐N and IL‐1β, with β‐actin serving as the reference for equal loading. (F‐I) Quantitation of NLRP3, Caspase‐1, GSDMD‐N and IL‐1β gray value (*n* = 3–4). The data are presented as the mean ± SD, one‐way ANOVA was employed followed by Tukey's post hoc test. *p < 0.05, **p < 0.01, ***p < 0.001, and ****p < 0.0001.

## Data Availability

All data used in this study are available upon reasonable request. All raw sequencing data have been deposited in NCBI SRA (SRA accession, SRP719896; BioProject accession, PRJNA1498347).
