# Supplementary material for: Histone H3K18 Lactylation Contributes to Perioperative Neurocognitive Disorder Through Immune Checkpoint Lymphocyte Activation Gene 3 Mediated Microglial Pyroptosis
Source: CNS Neurosci Ther. 2026 Jul 27;32(7):e71058. doi: 10.1002/cns.71058 (PMC13403156; doi:10.1002/cns.71058)
Supplement: Supplementary file 1 — Figure S1: Inhibition of glycolysis alleviates cognitive impairment induced by surgery in mice. (A) Timeline of 2‐DG intervention strategy and behavioral assessment. (B) Total distance in the OFT (n = 9). (C) Time spent in central zone (n = 9). (D) Numbers of entering the central zone (n = 9). (E) Percentage of investigation time of novel object in the NOR (n = 9). (F) After adaptation on the first day and exploration of two identical objects on the second day, a new object was replaced on the third day, and representative trajectories in the NOR were shown. (G, H) Total distance and latency in Barnes maze acquisition training (n = 9). (I) Typical trajectories within the probe trial procedure. (J) Total distance in probe trial latency (n = 9). (K) Latency time in probe trial (n = 9). (L) Mean velocity of movement (n = 9). The data are presented as the mean ± SD, one‐way ANOVA was employed followed by Tukey's post hoc test. *p < 0.05, **p < 0.01. # p < 0.05, ## p < 0.01, Sur + 2‐DG group vs. Sur group. Figure S2: Lag3 expression of microglia in mice hippocampus after surgery and after treatment with 2‐DG. (A) Mouse hippocampi were collected at 12 h, 24 h, 48 h, 72 h post‐surgery and after 2‐DG treatment, followed by WB and IF analysis of Lag3 expression in microglia. (B) Representative immunoblotting of Lag3 in mice hippocampus after surgery, with β‐actin used for normalization. (C) Quantitation of Lag3 gray value (n = 4). (D) Hippocampal levels of histone Pan‐Kla and H3K18la after treatment with 2‐DG, with histone H3 used for normalization. (E, F) Quantitation of Pan‐Kla and H3K18la gray value (n = 3). (G) Representative immunoblotting of Lag3 in mice hippocampus after treatment with 2‐DG, with β‐actin used for normalization. (H) Quantitation of Lag3 gray value (n = 3). (I) Representative fluorescence images of Lag3 in Iba‐1+cells in the hippocampus. (J) Lag3+/Iba‐1+ cell proportion (n = 3). The images include scale indicators of 50 μm for size reference. The data a [file CNS-32-e71058-s001.docx]

**Supplementary Figures**

**Histone H3K18 lactylation contributes to perioperative neurocognitive disorder through immune checkpoint Lymphocyte activation gene 3 mediated microglial pyroptosis**

**Chenglong Li^1^, Xi Gou^1^, Yingying Zhao^1^, Lina Zhang^1^, Shuai Liu^1^, Shenhui Deng^1^, Qi Li^1^ and Sihua Qi^1*^**

^1^Department of Anesthesiology, The Fourth Affiliated Hospital of Harbin Medical University, Harbin 150001, Heilongjiang, China.

^*^Corresponding author: Sihua Qi, MD, PhD, Department of Anesthesiology, The Fourth Affiliated Hospital of the Harbin Medical University, 37 Yiyuan Road, Harbin, Heilongjiang, China

Email: qisihua2007@163.com

**Supplementary Figure 1**


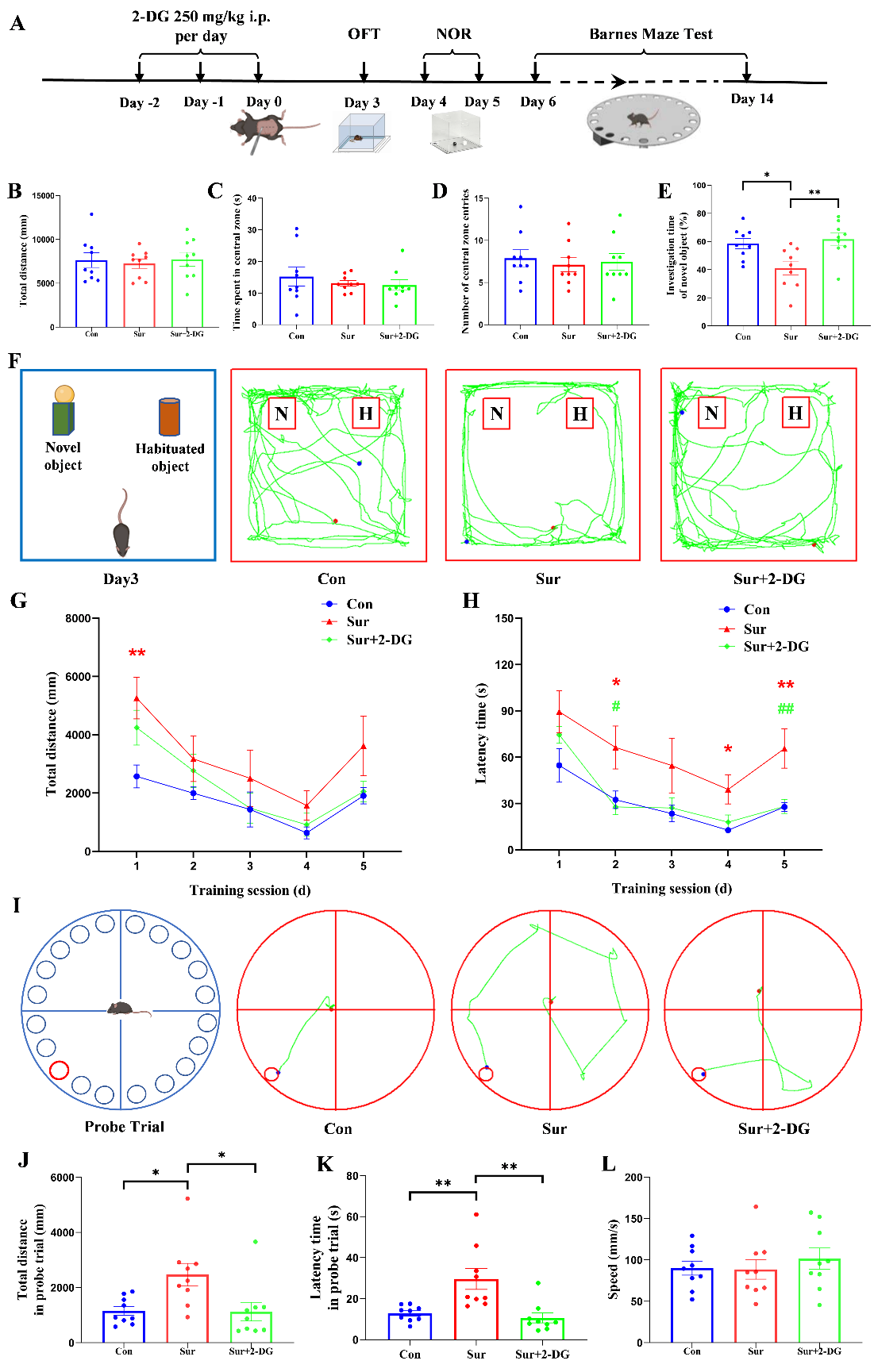


**Supplementary Figure 1. Inhibition of glycolysis alleviates cognitive impairment induced by surgery in mice. (A)** Timeline of 2-DG intervention strategy and behavioral assessment. **(B)** Total distance in the OFT (n = 9). **(C)** Time spent in central zone (n = 9). **(D)** Numbers of entering the central zone (n = 9). **(E)** Percentage of investigation time of novel object in the NOR (n = 9). **(F)** After adaptation on the first day and exploration of two identical objects on the second day, a new object was replaced on the third day, and representative trajectories in the NOR were shown. **(G, H)** Total distance and latency in Barnes maze acquisition training (n = 9). **(I)** Typical trajectories within the probe trial procedure. **(J)** Total distance in probe trial latency (n = 9). **(K)** Latency time in probe trial (n = 9). **(L)** Mean velocity of movement (n = 9). The data are presented as the mean±SD,one-way ANOVA was employed followed by Tukey's post-hoc test. ^*^*p* < 0.05, ^**^*p* < 0.01. ^#^*p* < 0.05, ^##^*p* < 0.01, Sur+2-DG group vs. Sur group.

**Supplementary Figure 2**


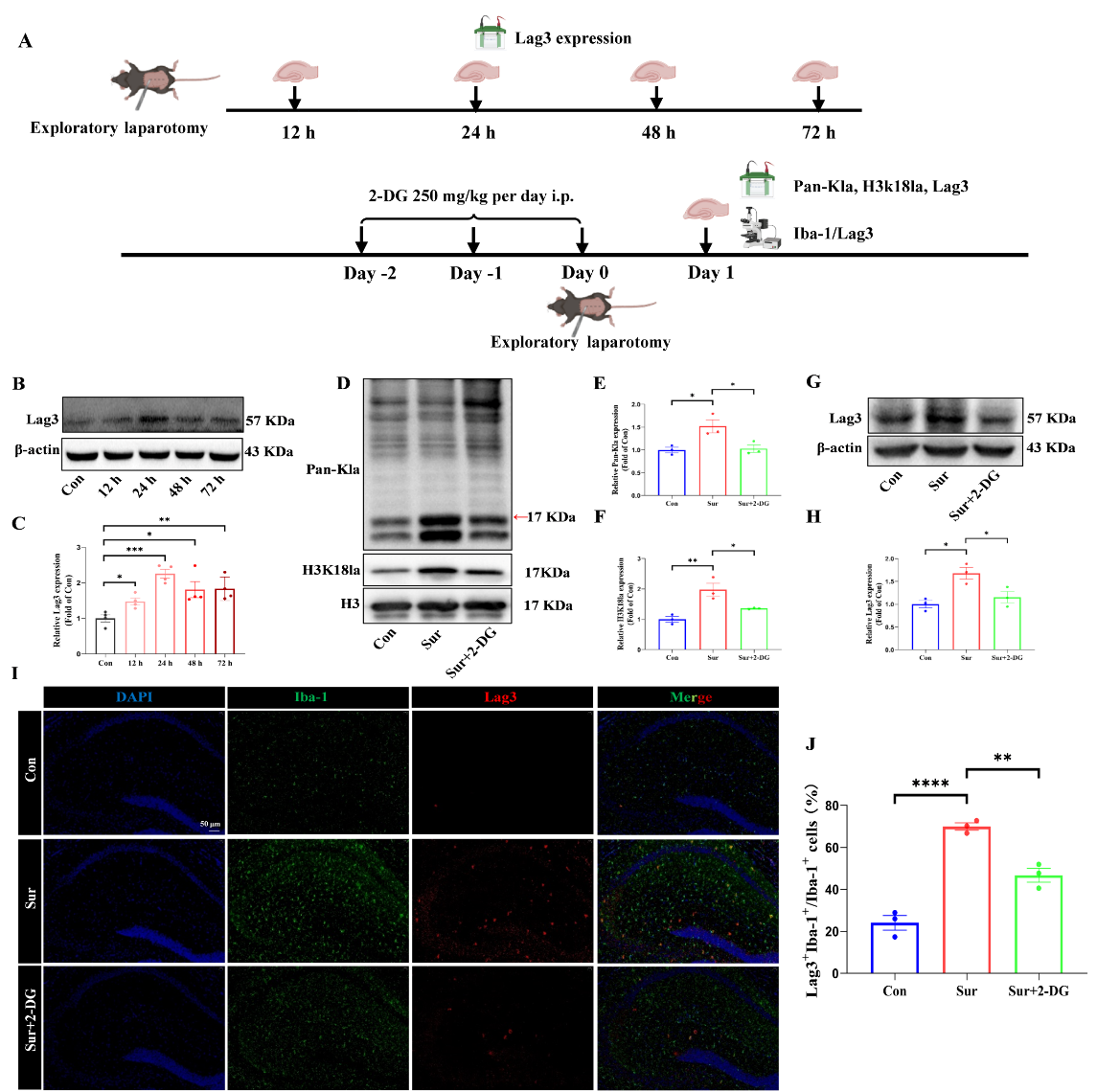


**Supplementary Figure 2. Lag3 expression of microglia in mice hippocampus after surgery and after treatment with 2-DG. (A)** Mouse hippocampi were collected at 12 h, 24 h, 48 h, 72 h post-surgery and after 2-DG treatment, followed by WB and IF analysis of Lag3 expression in microglia. **(B)** Representative immunoblotting of Lag3 in mice hippocampus after surgery, with β-actin used for normalization. **(C)** Quantitation of Lag3 grey value (n=4). **(D)** Hippocampal levels of histone Pan-Kla and H3K18la after treatment with 2-DG, with histone H3 used for normalization. **(E, F)** Quantitation of Pan-Kla and H3K18la grey value (n=3). **(G)** Representative immunoblotting of Lag3 in mice hippocampus after treatment with 2-DG, with β-actin used for normalization. **(H)** Quantitation of Lag3 grey value (n=3). **(I)** Representative fluorescence images of Lag3 in Iba-1^+^cells in the hippocampus. **(J)** Lag3^+^/Iba-1^+^ cell proportion (n=3). The images include scale indicators of 50 µm for size reference. The data are presented as the mean±SD, one-way ANOVA was employed followed by Tukey's post-hoc test. ^*^*p* < 0.05, ^**^*p* < 0.01, ^***^*p* < 0.001, ^****^*p* < 0.0001.

**Supplementary Figure 3**

**
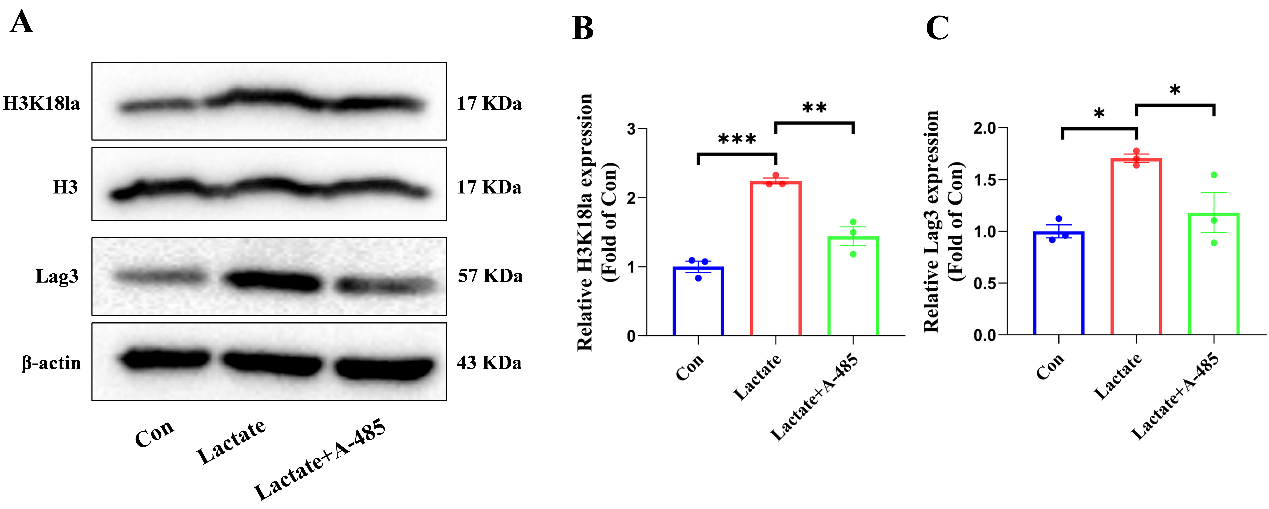
**

**Supplementary Figure 3. Inhibition of p300 by A-485 abolishes lactate-induced upregulation of H3K18la and Lag3 in BV2 cells. (A)** Representative immunoblotting of H3K18la and Lag3 in BV2 cells, with histone H3 or β-actin used for normalization. **(B, C)** Quantitation of H3K18la and Lag3 grey value (n=3). The data are presented as the mean±SD, one-way ANOVA was employed followed by Tukey's post-hoc test. ^*^*p* < 0.05, ^**^*p* < 0.01, ^***^*p* < 0.001.

**Supplementary Figure 4**

**
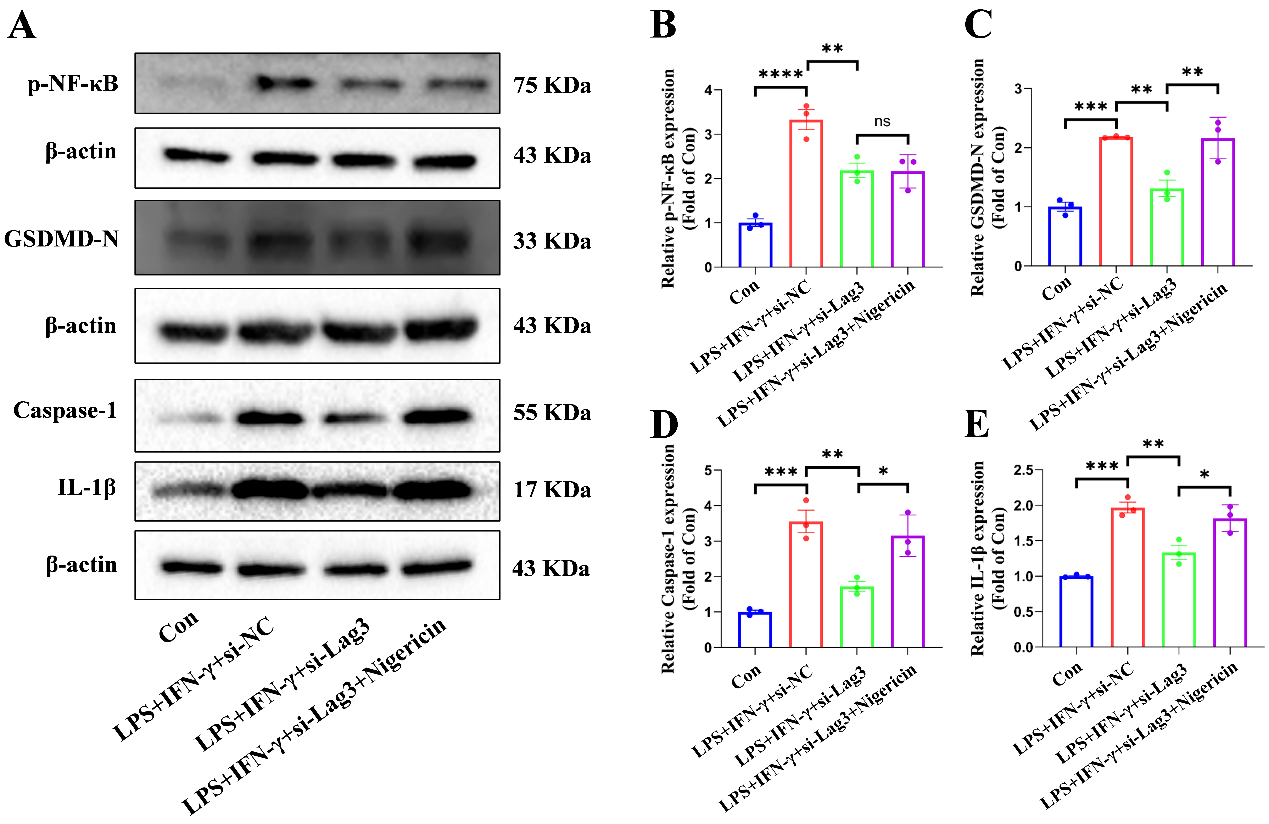
**

**Supplementary Figure 4. NLRP3 agonist nigericin reverses Lag3 knockdown-mediated inhibition of LPS/IFN-γ-induced pyroptosis in BV2 cells. (A)** Representative immunoblotting of p-NF-κB, GSDMD-N, Caspase-1 and IL-1β in BV2 cells, with β-actin used for normalization. **(B-E)** Quantitation of p-NF-κB, GSDMD-N, Caspase-1 and IL-1β grey value (n=3). The data are presented as the mean±SD, one-way ANOVA was employed followed by Tukey's post-hoc test. ^*^*p* < 0.05, ^**^*p* < 0.01, ^***^*p* < 0.001, ^****^*p* < 0.0001.

**Supplementary Figure 5**


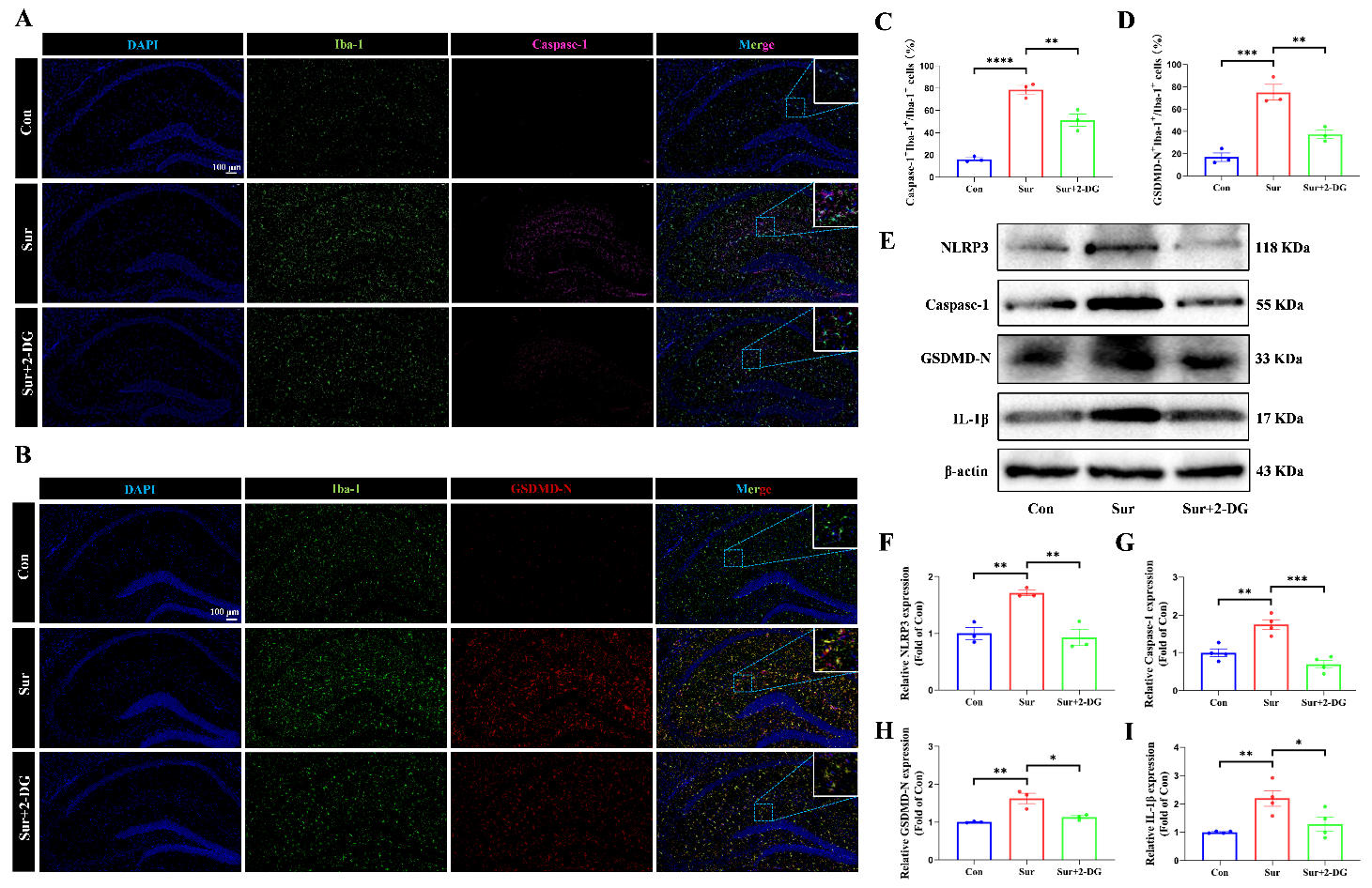


**Supplementary Figure 5. Inhibition of glycolysis reduces surgery-induced microglial pyroptosis in the mouse hippocampus. (A, B)** Representative fluorescence images of Caspase-1 and GSDMD-N expressions in Iba-1^+^ cells in the hippocampus. **(C)** Caspase-1^+^/Iba-1^+^ cell proportion (n=3). **(D)** GSDMD-N^+^/Iba-1^+^ cell proportion (n=3). **(E)** Representative immunoblotting of NLRP3, Caspase-1, GSDMD-N and IL-1β, with β-actin serving as the reference for equal loading. **(F-I)** Quantitation of NLRP3, Caspase-1, GSDMD-N and IL-1β grey value (n=3-4). The data are presented as the mean±SD, one-way ANOVA was employed followed by Tukey's post-hoc test. ^*^*p* < 0. 05, ^**^*p <* 0.01, ^***^*p* < 0.001, and ^****^*p* < 0.0001.
